# Novel Bunyavirus in Domestic and Captive Farmed Animals, Minnesota, USA

**DOI:** 10.3201/eid2002.131360

**Published:** 2014-02

**Authors:** Roger S. Nasci, Aaron C. Brault, Amy J. Lambert, Harry M. Savage

**Affiliations:** Centers for Disease Control and Prevention, Fort Collins, Colorado, USA

**Keywords:** bunyavirus, phlebovirus, Heartland virus, Heartland-like virus, hrtv, severe fever with thrombocytopenia syndrome virus, sftsv, animals, infection, zoonoses, viruses, Minnesota, USA, large mammals, novel bunyavirus

**To the Editor**: Xing et al. (*1*) conclude that evidence of infection with a severe fever with thrombocytopenia syndrome (SFTS)–like virus or Heartland-like virus (HRTV) was found in many captive large mammals from much of Minnesota, raising the specter of widespread distribution of a novel pathogen. Although it is likely that HRTV can be found beyond the areas in northwestern Missouri, where it was discovered (*2*), we contend that this conclusion is not substantiated by the data presented by Xing et al., which were generated by an assay that was developed to diagnose SFTS virus infections in China (*1*,*3*). The study used an ELISA developed for an SFTS virus recombinant nucleocapsid protein that detects SFTS-reactive antibodies (*3*). The conclusions reached by Xing et al. are based on the assumption that the SFTS assay developed in China will cross-react with HRTV antibodies (*1*). This assumption remains unsupported because the SFTS assay has not been evaluated for cross-reaction with antibodies to other non-SFTS members of the genus *Phlebovirus* (*1*,*3*). In addition, it is well recognized that serologic tests, like the ELISA, are often group reactive (*4*), requiring neutralization tests to confirm antibody presence and provide specificity. Alternative explanations include the possibility that positive results from testing by Xing et al. may have been caused by cross-reaction with antibodies directed against other known tick-associated phleboviruses endemic to North America, such as Lone Star virus (*5*), which is not known to be pathogenic. In the absence of confirmatory data generated by an independent method, the report by Xing et al. (*1*) should be considered speculative. Reports suggesting substantial expansion in the geographic range of a pathogenic organism should be based on rigorously validated laboratory methods.
